# Platelets, Thrombocytosis, and Ovarian Cancer Prognosis: Surveying the Landscape of the Literature

**DOI:** 10.3390/ijms21218169

**Published:** 2020-10-31

**Authors:** Demetra H. Hufnagel, Gabriella D. Cozzi, Marta A. Crispens, Alicia Beeghly-Fadiel

**Affiliations:** 1 Vanderbilt University School of Medicine, 2209 Garland Avenue, Nashville, TN 37240, USA; demetra.h.hufnagel@vanderbilt.edu (D.H.H.); gabriellacozzi@uabmc.edu (G.D.C.); 2 Department of Obstetrics and Gynecology, Division of Gynecologic Oncology, Vanderbilt University Medical Center, 1161 21st Avenue South, Nashville, TN 37232, USA; marta.crispens@vumc.org; 3 Vanderbilt-Ingram Cancer Center, 1301 Medical Center Drive, Nashville, TN 37232, USA; 4 Department of Medicine, Division of Epidemiology, Vanderbilt University Medical Center, 2525 West End Avenue, Nashville, TN 37203, USA

**Keywords:** platelets, thrombocytosis, ovarian cancer, prognosis

## Abstract

Platelets are critical components of a number of physiologic processes, including tissue remodeling after injury, wound healing, and maintenance of vascular integrity. Increasing evidence suggests that platelets may also play important roles in cancer. In ovarian cancer, thrombocytosis, both at the time of initial diagnosis and at recurrence, has been associated with poorer prognosis. This review describes current evidence for associations between thrombocytosis and ovarian cancer prognosis and discusses the clinical relevance of platelet count thresholds and timing of assessment. In addition, we discuss several mechanisms from in vitro, in vivo, and clinical studies that may underlie these associations and recommend potential approaches for novel therapeutic targets for this lethal disease.

## 1. Introduction

Circulating anucleate cytoplasmic fragments of megakaryocytes, platelets play critical and well-characterized roles in wound healing and maintenance of vascular integrity by adhering, activating, and aggregating at sites of vascular injury to form blood clots and stop bleeding [1]. While unprovoked thrombosis has long been associated with an increased risk of malignancy, as first described by Armand Trousseau in 1865, growing evidence has demonstrated an expanded role for platelets in the promotion of inflammation, tumorigenesis, and cancer metastasis [2,3,4,5]. Although platelets are anucleate, they have a number of cell surface receptors by which they interact with their environment, including integrins, selectins, immunoglobin receptors, and glycoproteins; they also contain a rich assortment of growth factors and mitogenic proteins in granules that they release following activation [1,6,7,8,9]. High platelet count, or thrombocytosis, is most often defined as >400,000 platelets per microliter and has been widely associated with poor cancer prognosis in a variety of solid tumors, including non-small cell lung cancer, advanced breast cancer, pancreatic cancer, colorectal cancer, and gastric cancer [10,11,12,13,14,15,16,17,18]. Although the mechanisms underlying associations between paraneoplastic thrombocytosis and cancer progression have yet to be fully elucidated, there is strong evidence for reciprocal interactions between tumor growth and platelet production and activation; accumulating data suggest that platelets are not only a biomarker of disease burden, with increased levels at diagnosis falling after primary treatment and again rising at recurrence, but also actively contribute to disease progression [19,20].

Ovarian cancer is the most lethal of the gynecologic cancers, with recent estimates of a relative 5-year survival of 48%, often due to diagnosis at late stage given non-specific symptoms and lack of sensitive population screening tools [21,22,23,24]. Although overall mortality from cancer has dramatically declined over recent decades, mortality from ovarian cancer has largely remained unchanged [21]. A better understanding of the pathogenesis of this disease is necessary for the development of additional therapeutic strategies. As with many other cancers, thrombocytosis is common during preoperative evaluation of women with epithelial ovarian cancer, affecting approximately 30% of patients [25]. Thrombocytosis has also been found to be associated with cancer risk in the setting of an adnexal mass, as well as characteristics of aggressive malignancy, including advanced stage, higher grade, larger volume of ascites, extensive residual disease following debulking, and chemoresistance [26,27,28,29,30,31,32,33,34,35,36,37,38]. A study of 30-day post-surgical outcomes among 1072 ovarian cancer cases demonstrated an association between thrombocytosis and major complications [39]. Moreover, thrombocytosis has been demonstrated to be a prognostic factor for multiple outcomes in numerous studies (Table 1) with significant associations from both univariate and multivariate analyses, suggesting independent associations with disease outcomes. The mechanisms behind these associations are now under investigation; therapeutics with anti-platelet activity, such as non-steroidal anti-inflammatory drugs (NSAIDs) including aspirin, have been associated with better prognosis in epidemiologic studies, and anti-platelet therapies are now being investigated in the ovarian cancer clinical setting [40,41,42,43].

Despite a large body of evidence supporting a role for thrombocytosis in ovarian cancer prognosis, clinical guidelines for monitoring platelets as a prognostic indicator to inform conversations with patients have yet to be determined. While prior meta-analyses and reviews have supported associations between pretreatment thrombocytosis and ovarian or gynecologic cancer survival, in this review we discuss timing of platelet count measurements and thresholds for thrombocytosis with regard to survival among women with epithelial ovarian cancer, with consideration of statistical adjustment and other important methodological differences [44,45]. We also describe a number of mechanisms potentially underlying these associations that suggest platelets are active contributors to the progression of disease and may present novel therapeutic targets in ovarian cancer.

## 2. Pretreatment Thrombocytosis and Associations with Survival

To the best of our knowledge, 29 studies have evaluated the prognostic utility of thrombocytosis for ovarian cancer in the preoperative setting—either at primary staging and/or cytoreduction—or pretreatment. We focus primarily on 27 that conducted Kaplan–Meier and/or Cox proportional hazards regression to estimate associations with progression-free survival (PFS), disease-free survival (DFS), disease-specific survival (DSS), and/or overall survival (OS). To facilitate review of studies with varied methodologies, we have presented the relevant literature divided by thrombocytosis threshold (below 400, 400, above 400, and multiple thresholds or continuous measures), with the timeframe within which platelet counts were included also being considered.

### 2.1. Pretreatment Thrombocytosis and Associations with Survival: Timing of Platelet Count Measurement

Among 27 studies that have assessed thrombocytosis prior to treatment in relation to ovarian cancer survival, only 12 (44%) provide the specific time frame within which platelet counts were included; these have ranged from the date of diagnosis to up to 8 weeks or 3 years prior to diagnosis [26,46]. Many studies have used a time frame of within 7 or 14 days of initial staging and/or cytoreductive surgery, but whether the date of diagnosis was included has been rarely stated. The remaining studies have examined platelet counts from either the “preoperative” or “pretreatment” setting without any further specification.

### 2.2. Pretreatment Thrombocytosis and Associations with Survival: Thresholds Below 400

Several small studies employing a threshold below 400 × 10^9^/L for thrombocytosis in relation to ovarian cancer prognosis have had significant findings. In a small study of 97 patients in Poland with platelet counts measured 1 day prior to staging and/or debulking surgery, thrombocytosis (≥350) was found to be a negative prognostic factor for OS, although no regression was conducted [47]. In a study of 91 advanced stage serous cases in Switzerland, thrombocytosis (>350) was an independent negative prognostic factor for two-fold worse PFS and OS, even after multivariable regression including adjustment for age, stage, menopausal status, CA-125, and optimal tumor debulking [27]. In a study with 104 patients in China with recurrent disease, pre-primary treatment thrombocytosis (≥300) conferred significantly worse PFS and OS, even when regression models included adjustment for age, stage, grade, residual disease after cytoreduction, and reduced platelet count after completion of first-line treatment [48]. Similarly, in a study of 190 patients in China with platelet measurements up to 7 days prior to treatment, thrombocytosis (>300) was significantly associated with approximately 50% worse 3-year PFS and OS in models that included adjustment for age, stage, grade, optimal debulking, timing of surgery, chemotherapy, clotting factors, and thrombotic events [32].

### 2.3. Pretreatment Thrombocytosis and Associations with Survival: Thresholds of 400

Multiple studies have employed a threshold of 400 × 10^9^/L for thrombocytosis in relation to ovarian cancer prognosis, with most reporting significant findings. An early study of 70 patients in Israel found that those with thrombocytosis had worse OS [49], while a study of 130 patients in Austria found no difference, although their mean follow-up was only two years [50]. Among 136 cases from China, initially significant associations with PFS and OS were attenuated after regression models included adjustment for stage, cytoreduction, chemotherapy, CA-125, and plasma fibrinogen level, which was significantly correlated with platelet count (r = 0.45) [51]. In a United States (U.S.) study with platelet measurements from up to 14 days prior to diagnosis, thrombocytosis was significantly associated with two-fold worse OS when multivariable regression models were limited to 144 patients with late stage (III and IVA) disease [30]. Similarly, in a study of 179 women in Korea with advanced stage (III and IV) disease, thrombocytosis based on measurements from up to 7 days prior to diagnosis was associated with approximately three-fold worse OS, even after adjustment for tumor histology, platelet count following chemotherapy, and CA-125 following primary chemotherapy [33]. Among 280 ovarian cancer cases in Japan, associations with PFS and OS were significant in both unadjusted and multivariable adjusted regression models [52], and among 292 cases in Turkey, OS was two-fold worse for those with thrombocytosis based on measurements from within 14 days of surgery [53]. In a large study of 816 patients in China, thrombocytosis was associated with significantly increased hazards of disease progression and death, even when multivariable regression models included adjustment for age, stage, grade, ascites, optimal debulking, and anemia [54]. The largest study to date included 1308 cases with clear cell or serous ovarian cancer from 10 academic institutions; thrombocytosis was an independent predictor of both PFS and OS, even in regression models that included adjustment for venous thromboembolism (VTE) [55]. In addition, two studies have assessed thrombocytosis in relation to ovarian cancer survival in combination with other factors. Among 108 advanced stage (IV) patients in China, those with thrombocytosis and/or elevated CA-125 had significantly worse PFS and OS [56], while among 182 patients in China, those with thrombocytosis and a high platelet mean aggregation rate (MAR) had more than three-fold higher hazards of death [31].

### 2.4. Pretreatment Thrombocytosis and Associations with Survival: Thresholds Above 400

Multiple studies have employed a threshold of 450 × 10^9^/L for pretreatment thrombocytosis, but in studies that have used only this threshold, specific timings of platelet measurements have not been described and findings have been mixed. In a small study of 72 ovarian cancer cases in Nigeria, thrombocytosis was significantly associated with worse PFS and OS within 3 years [57]. A multi-institution study that included 341 patients with recurrent or progressive disease reported that thrombocytosis at diagnosis was a negative prognostic factor for both PFS and OS, although proportional hazards regression was not conducted [34]. Some of these patients were also included in a larger study of 619 total patients from four academic medical centers in the U.S. where thrombocytosis was found to be an independent predictor of survival, with 80% worse OS, even after statistical adjustment for age, stage, grade, histologic subtype, and cytoreduction [25]. This finding was replicated among 578 cases from the Mayo Clinic, where thrombocytosis was associated with approximately 40% worse DFS and OS, although significance was attenuated when Eastern Cooperative Oncology Group (ECOG) performance status and American Society of Anesthesiologists (ASA) scores were included in the regression models [29]. Among 874 high-grade serous ovarian cancer cases in China, thrombocytosis was significantly associated with stage, ascites volume, residual disease after debulking, and chemosensitivity, but not PFS or OS in Kaplan–Meier survival analysis [28]. However, hyperfibrinogenemia (>4g/L) was significantly associated with survival, leading the authors to conclude that while thrombocytosis reflects tumor burden and thus influences treatment outcomes, only fibrinogen was a useful prognostic factor for high-grade serous ovarian cancer [28].

### 2.5. Pretreatment Thrombocytosis and Associations with Survival: Multiple Thresholds and Continuous Measures

Studies that have compared multiple thresholds for thrombocytosis or evaluated continuous measures of platelet counts in the context of ovarian cancer survival are less common. One study with 120 cases from Thailand started with an *a priori* threshold of 400, but then conducted a Receiver Operating Curve (ROC) analysis on the relationship between thrombocytosis and stage, finding that 305 was best able to differentiate between early and advanced disease [58]. When both thresholds (305, 400) were evaluated with regard to survival in a cohort of 74 patients with advanced epithelial ovarian cancer, only platelet counts above the 305 threshold from up to 14 days before primary surgery were significantly associated with worse overall survival in Kaplan–Meier analysis, but no proportional hazards regression was conducted [58]. A study of 308 patients in Japan used ROC analysis to define an optimal threshold for preoperative thrombocytosis and disease-specific survival; if the lowest platelet count between diagnosis and treatment was above 427 × 10^9^/L, then patients had a nearly two-fold increased risk of death due to ovarian cancer, even following adjustment for known clinical covariates, such as age, stage, metastasis, and cytoreduction [59]. However, this association was attenuated when the analysis was stratified by nutritional status, based on serum albumin and lymphocyte count, which was inversely correlated with platelet count (r = −0.39) [59]. A cohort study in Denmark with 224 women who developed ovarian cancer used platelet counts from up to 3 years before diagnosis and defined thrombocytosis as mild (>400 × 10^9^/L) or severe (>550 × 10^9^/L); while mild was not significantly associated, severe thrombocytosis conferred a more than two-fold increased risk of cancer-specific and overall mortality [46]. Our prior study of 304 cases from the Vanderbilt University Medical Center (VUMC) compared three thresholds (350, 400, and 450), with increasing timeframes (on the date of, and 1, 2, 4, and 8 weeks prior to diagnosis); with the exception of the lowest threshold on the date of diagnosis, all other associations were significant, with estimates ranging from 50% to a two-fold increased risk of death [26]. A smaller study of 171 ovarian cancer cases from China supported these findings, comparing thresholds of 300, 350, and 400 in Kaplan–Meier analysis; all three thresholds were associated with worse OS, and ROC analysis suggested an optimal threshold of >327.5 × 10^9^/L for predicting overall survival [60]. A larger study of 498 patients in Austria evaluated thrombocytosis using both a ≥450 × 10^9^/L threshold and a continuous measure; although initially significant, neither remained associated with OS after statistical adjustment for age, stage, grade, histologic subtype, residual disease, and serum sodium measures [61]. Finally, among 118 advanced stage cases from the Netherlands, each unit increase in platelet count within a week prior to surgery conferred a 0.2% increased risk of disease progression or death [62].

### 2.6. Pretreatment Thrombocytosis and Associations with Survival: Multivariable Adjustment

The relationship between pretreatment thrombocytosis and ovarian cancer survival seems to be robust, as regression models from studies where significance was not attenuated have adjusted for a multitude of clinical covariates, including age, stage, grade, histologic subtype, chemotherapy, chemosensitivity, optimal tumor debulking/residual disease after cytoreduction, ascites volume, CA-125, menopausal status, anemia, and thrombotic events/VTE [25,26,27,30,32,33,48,52,53,54,55,57,59,62]. However, multivariable adjustment has resulted in attenuation of the association between thrombocytosis and ovarian cancer survival in some cases, such as when adjustment included plasma fibrinogen level, serum sodium measures, or ECOG and ASA scores [29,51,61]. Attenuation of an association after multivariable adjustment may indicate a false negative finding, possibly due to highly collinear variables being included in a regression model, or an inadequate sample size. Alternatively, thrombocytosis may not be an independent prognostic factor for ovarian cancer, although so many studies reporting an independent association due to chance (i.e., false positive findings) is unlikely.

## 3. Thrombocytosis after Primary Treatment and Associations with Survival

Eight studies have evaluated the prognostic utility of thrombocytosis for ovarian cancer in the post-diagnostic period, most frequently focused on platelet count at the time of disease progression or recurrence. Timeframes ranged from before or after first-line chemotherapy to preoperative platelet counts for second-look laparotomy or secondary cytoreductive surgery for recurrent disease. Similar to pretreatment criteria, thrombocytosis thresholds ranged from 300 to 450 × 10^9^/L.

### 3.1. Thrombocytosis Following Chemotherapy and Associations with Survival

Three studies on thrombocytosis in ovarian cancer have included chemotherapy-related platelet counts. A study in China of 104 patients with recurrent disease evaluated not only pretreatment measures, but also those taken 14 days after completing chemotherapy and at the time of disease recurrence; in line with prior studies, they found that platelet levels generally declined at the end of primary therapy and rose again at disease recurrence [20,48]. The ratio between pretreatment and post-chemotherapy platelet counts was calculated, and a reduction of less than 25% was found to be an independent negative prognostic factor for survival with a greater than 2-fold increased risk of death even when pretreatment platelet count was adjusted for [48]. A study with 132 ovarian cancer patients in Germany evaluated platelet counts at diagnosis, after surgery, before and after chemotherapy, and at the time of recurrence if applicable; when the ratio of platelet counts before and after chemotherapy was assessed, a less than 25% reduction was found to be an independent negative prognostic factor for both PFS and OS [20]. Among 179 advanced stage (III and IV) cases in Korea, thrombocytosis (≥400 × 10^9^ platelets/L) after primary adjuvant chemotherapy was associated with significantly worse OS [33].

### 3.2. Thrombocytosis at Disease Recurrence and Associations with Survival

Three studies on thrombocytosis in ovarian cancer have examined platelet counts around the time of recurrence in relation to prognosis. A study of 107 patients in the U.S. demonstrated that preoperative thrombocytosis (≥350 × 10^9^ platelets/L) at the time of secondary cytoreductive surgery was significantly associated with suboptimal resection and worse OS in both unadjusted and adjusted analyses [63]. A study of 300 cases with recurrent disease who participated in three phase-III clinical trials (AGO-OVAR 2.2, AGO-OVAR 2.3, and AGO-OVAR 2.9) found that thrombocytosis (≥400 × 10^9^ platelets/L) prior to second-line chemotherapy was associated with chemoresistance and worse OS; however, when regression models included ECOG performance status, significance of the association was attenuated [64]. Notably, this study excluded cases who underwent secondary cytoreductive surgery before randomization to trial, as thrombocytosis may be associated with acute inflammation and stress-response following surgical procedures. A study of 787 women with clear cell or serous ovarian cancer from ten academic institutions in England, Japan, and the U.S., found that thrombocytosis (≥400 × 10^9^ platelets/L) at disease recurrence or progression was an independent predictor for 60% decreased two-year OS, even after age, CA-125, histologic subtype, and VTE were adjusted for [55].

Although time-to-event analyses were not conducted, two additional studies have evaluated post-diagnosis platelet counts. A small prospective study used ROC analysis to identify an optimal threshold of 380 × 10^9^/L to differentiate between patients with and without disease recurrence or progressive disease at the time of second-look laparotomy; among 37 patients, those with higher platelet counts after completing chemotherapy were also more likely to have evidence of disease progression at the time of second-look surgery [65]. Similarly, a retrospective study identified 96 cases from three academic medical centers in the U.S. with platelet data spanning initial diagnosis, primary treatment, and diagnosis of disease recurrence; counts were found to decrease during primary therapy and again increase at disease recurrence [34]. Further, among a subset of 20 cases matched on clinical covariates, ten with a durable response were more likely than ten with treatment refractory disease to have a normalized platelet count at the end of primary chemotherapy [34].

## 4. Mechanisms Underlying Associations of Thrombocytosis with Disease Progression

**Figure 1:**
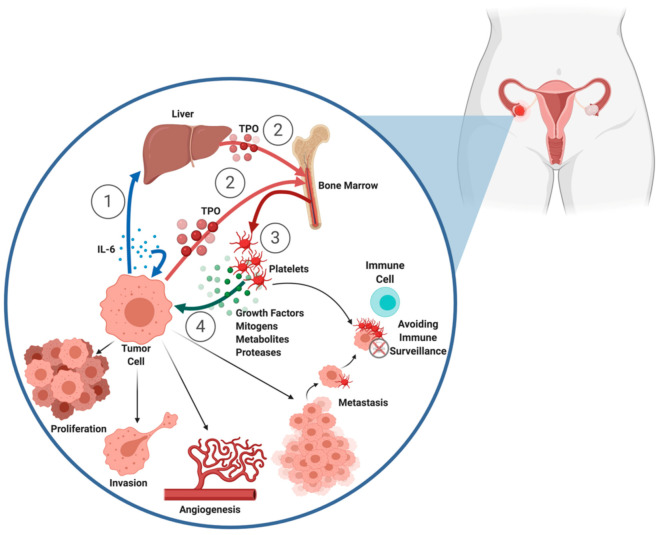
Contributions of platelets to ovarian cancer progression. (**1**) Tumor cells produce the cytokine interleukin-6 (IL-6). (**2**) IL-6 drives production of thrombopoietin (TPO) directly from tumor cells as well as the liver. (**3**) TPO stimulates production and release of platelets from bone marrow. (**4**) Platelets support cancer cell proliferation, invasion, angiogenesis, migration, and metastasis through release of growth factors, mitogens, metabolites, and proteases. They may also act as sites of seeding for metastasis and form aggregates with tumor cells to shield them from immune surveillance. Figure 1 is an original figure that was created by the authors using BioRender.com.

While a large body of epidemiologic literature suggests that platelets are associated with ovarian cancer prognosis, mechanistic studies evaluating the role of platelets in modulating disease progression provide evidence that this association is not purely correlative; both preclinical and clinical studies demonstrate that platelets and cancer cells influence each other in a bidirectional manner, wherein malignant neoplasms support thrombocytosis and increased platelets drive tumorigenesis and metastasis (Figure 1) [3].

### 4.1. Cytokine-Driven Development of Thrombocytosis in Ovarian Cancer

Prior studies have established that paraneoplastic thrombocytosis is driven in part by cancer cell-mediated release of interleukin-6 (IL-6), which stimulates the production of hepatic-derived and/or cancer-cell derived thrombopoietin to drive platelet overproduction [25,66,67]. In orthotopic and syngeneic murine models of ovarian cancer, increases in disease burden are associated with concomitant increases in IL-6 and platelet counts [25]. Critically, in these studies, blockage of IL-6 rescued mice from cancer-associated thrombocytosis, and murine models lacking a hepatic IL-6 receptor did not have elevations in platelet count associated with tumor growth, corroborating the major role IL-6 plays in mediating cancer-related thrombocytosis. In human samples, ovarian tumor and plasma expression of IL-6 was likewise significantly associated with plasma thrombopoietin levels as well as thrombocytosis. There is some evidence that in cancers with *PIK3CA* mutations, such as endometrioid and clear cell ovarian cancers, upregulation of nuclear factor-kappaB (NF-κB) pathways may drive the expression of IL-6, perhaps relating known associations between increased NF-κB expression and poor prognosis in ovarian cancer to mechanisms underlying associations with thrombocytosis [68,69,70,71,72,73,74].

### 4.2. Contributions of Platelets to Ovarian Cancer Progression: In Vitro and In Vivo Studies

A number of in vitro and in vivo studies have demonstrated that exposure to platelets drives anti-apoptotic, pro-invasive, and pro-angiogenic activity in human ovarian cancer cells. This is perhaps unsurprising given that platelets contain a variety of growth factors, mitogens, metabolites, and proteases in their granules and lysosomes, including platelet-derived growth factor (PDGF), transforming growth factor (TGF), vascular endothelial growth factor (VEGF), metalloproteinases, collagenases, and elastases [1,7,8,9].

Platelets may stimulate cells within the tumor microenvironment, either directly or indirectly through paracrine mechanisms, driving further production of pro-tumorigenic products and phenotypic shifts [8]. Holmes et al. demonstrated a dose-dependent increase in invasive activity of human ovarian cancer SKOV3 cells with exposure to activated platelets, which was decreased with platelet inhibition by prostaglandin E1 [75]. A protective effect against apoptosis from co-culture with platelets was similarly shown in A2780, HeyA8, and SKOV3-ip1 ovarian cancer cell lines, and studies of both human (SKOV3, OVCAR5) and murine (ID8, 2C6) ovarian cancer cell lines have demonstrated direct proliferative effects of platelets on cancer cells mediated through the TGFβ receptor [34,76]. Co-culture of ovarian cancer cells with platelet microparticles or platelets furthermore increases the expression of tumor protein products associated with epithelial–mesenchymal transition [60,77]. Moreover, addition of platelet antagonists to co-cultures decreases ovarian cancer cell invasive activity [77]**.** These effects have been seen with both inhibitors of the platelet-activating adenosine 5′-diphosphate receptor (P2Y_12_) and aspirin, which inhibits platelet cyclooxygenase to block formation of thromboxane, thereby preventing platelet adhesion and aggregation. Bottsford-Miller et al. leveraged murine models to investigate the therapeutic potential of anti-platelet therapies in ovarian cancer; platelet depletion—with and without chemotherapy—was associated with significant reductions in tumor weight, while platelet transfusion significantly increased tumor weight in orthotopic nude mice. Intriguingly, when transfused platelets were pretreated with aspirin or a direct thromboxane inhibitor, the pro-tumorigenic effects of platelet transfusion were blocked; while some preclinical evidence suggests that aspirin may have intrinsic anti-tumor activity, this specifically only occurred in the presence of platelets, indicating that these results were mediated by effects on platelets [34,78,79,80]. Inhibition of the P2Y_12_ platelet receptor has correspondingly been shown to reduce platelet-associated tumor growth in murine models of ovarian cancer [81,82]. In a similar study of the A2780ip2 mouse model, treatment with anti-platelet antibodies resulted in significant reductions of tumor proliferation and microvessel density, as well as substantial tumor necrosis [25].

While platelets activated by VEGF have been shown to participate in extravasation and metastasis of tumor cells, and platelet-derived VEGF in turn promotes angiogenesis by driving further recruitment of endothelial cells, platelets also drive angiogenesis in the ovarian cancer tumor microenvironment [83]. In vitro studies have demonstrated that exposure of ovarian cancer cell lines to activated platelets is associated with a significant increase in VEGF secretion into cellular media [75]. In murine models of ovarian cancer, endothelial markers VEGF and CD31 were found to co-localize with platelets, and co-culture of human ovarian cancer cell lines with platelets increases secretion of several pro-angiogenic factors [84,85]. Intriguingly, this preclinical evidence suggests that metformin may abrogate the pro-angiogenic activity of platelets in ovarian cancer [85]. Platelets may also play a role in tumor growth following cessation of anti-angiogenic therapy; withdrawal of anti-VEGF therapies is associated with accelerated tumor growth and concurrent tumor platelet-infiltration, while platelet depletion mitigates these effects in vivo [86]. In addition to promoting tumor angiogenesis and proliferation, formation of tumor-platelet aggregates also serves to shield cancer cells from immune surveillance and shear stress in circulation [4,87].

### 4.3. Contributions of Platelets to Ovarian Cancer Progression: Ex Vivo Studies

Studies of ex vivo human-derived ovarian cancer cells isolated from ascitic fluid have also supported a pro-tumorigenic role of platelets in ovarian cancer. Co-culture of patient-derived ovarian cancer cells with platelets resulted in increased migration and phenotypic changes characteristic of stem-cell-like behavior and epithelial–mesenchymal transition [88]. Further, platelets serve as chemoattractants to tumor cells, suggesting they may act as sites for metastatic seeding, particularly in the peritoneal cavity [88,89]. Ultrastructure variations and increased aggregation in platelets isolated from patients with invasive ovarian cancer may further influence these associations; however, studies evaluating differences in platelet aggregation and activity in ovarian cancer patients have had inconsistent findings [90,91]. Genetic variations in androgen receptor among patients with epithelial ovarian cancer have also been implicated in associations of thrombocytosis with poor survival, as androgens augment platelet activation and activity [92].

### 4.4. Contributions of Platelets to Ovarian Cancer Progression: Clinical Data

Studies in the clinical setting have also suggested potential mechanisms underlying the association of thrombocytosis with worse ovarian cancer progression. Among a cohort of 150 patients with newly diagnosed epithelial ovarian cancer, plasma levels of IL-6 and thrombopoietin corelated with platelet counts and in a smaller cohort, blockage of IL-6 with the humanized anti-IL-6 antibody siltuximab resulted in sustained and significant reductions of platelet counts [25]. A small clinical trial of siltuximab in patients with platinum-resistant ovarian cancer further demonstrated periods of disease stabilization in approximately 40% of patients, and significant declines in plasma levels of relevant pro-tumorigenic cytokines including VEGF. However, platelet parameters were not assessed [93]. Recent studies demonstrating associations between expression of Rac1, which is involved in platelet activation and aggregation, epithelial–mesenchymal transition, invasion, vascularity, and ovarian cancer prognosis may provide further mechanistic insights to associations of thrombocytosis with survival [94,95,96]. This connection is particularly intriguing given the evidence that the R-enantiomer of ketorolac, a widely available drug, exhibits potent inhibition of Rac1 activity and growth of ovarian cancer cells, and peri-operative administration has been associated with a survival benefit in previous retrospective studies of patients with ovarian cancer [97,98,99]. While use of aspirin has been previously thought to be associated with a reduced risk of ovarian cancer due to inhibition of NF-κB and inflammatory pathways, epidemiologic data also suggests a role for post-diagnostic use of aspirin and other NSAIDs with anti-platelet activity in decreasing production of thromboxane, a mediator of platelet aggregation, and mitigating disease progression and survival [41,42,43,100,101,102,103,104].

Associations of thrombocytosis with VTE and pulmonary embolism (PE) have also been suggested to underlie associations with mortality among ovarian cancer patients. Although thrombocytosis is strongly associated with risk of thrombosis in cancer patients, suggesting that platelets play a sizeable role in that process, most but not all studies continue to demonstrate significant associations of elevated platelet count with poor prognosis after statistical adjustment for VTE and/or PE [2,32,55,105].

## 5. Conclusions: Incorporation of Platelets into the Clinic and Future Therapeutic Targets

This review provides strong evidence to support prior claims that thrombocytosis is an independent negative prognostic factor for progression-free and overall survival in epithelial ovarian cancer. A 2019 meta-analysis of 11 studies reported a summary Hazard Ratio (HR) of 1.72 (95% Confidence Interval (CI): 1.44–2.06) for OS and a summary HR of 1.45 (95% CI 1.32–1.59) for PFS [44]. Here, we have compiled information from 32 relevant studies (24 with only pretreatment and 8 that include platelet measures from after initial treatment) and compared and contrasted methodological details. Although studies using thresholds as low as >300 × 10^9^/L have demonstrated significantly decreased progression-free and overall survival, approximately 50% used >400 × 10^9^/L as a platelet count threshold to define thrombocytosis. Among 27 studies assessing pretreatment thrombocytosis at the time of primary therapy, 11 (41%) have assessed platelet levels within 14 days of operative diagnosis, while 15 (56%) have assessed platelets from the pretreatment or preoperative periods without further specification. Among 18 studies that have assessed independent associations with OS, DSS, or cancer-specific mortality and pretreatment thrombocytosis, 15 (83%) demonstrated significant associations with worse survival in multivariable adjusted models. Similarly, among 11 studies that have evaluated PFS or DFS in multivariable adjusted analyses, 10 (91%) demonstrated significant independent associations with thrombocytosis. Based on the published studies reviewed, we conclude that preoperative thrombocytosis in the setting of first-line treatment has a robust association with worse survival in epithelial ovarian cancer, and thrombocytosis prior to the date of primary surgery (most often within 14 days) is an indicator of increased risk of disease progression and mortality for patients with epithelial ovarian cancer. While future studies should seek to evaluate these questions prospectively, there is now an abundance of evidence demonstrating this association retrospectively across multiple centers.

Evaluating platelet counts throughout treatment and surveillance to monitor response to therapy and progression of disease may have clinical utility. Although only 8 studies to date have evaluated associations of post-treatment platelet counts with prognosis, they unanimously found either associations with recurrence, characteristics of aggressive disease, such as chemoresistance, or worse prognosis. Of note, 2 studies described significantly worse prognosis when post-treatment platelet levels decreased by less than 25% as compared to pretreatment levels. Furthermore, the only published prospective study assessing thrombocytosis and ovarian cancer prognosis suggested that thrombocytosis prior to second-look surgery may also predict disease progression [65]. The utility of thrombocytosis as a biomarker for monitoring disease progression and treatment response should be evaluated in additional clinical settings.

While platelets and tumor cells may synergistically affect each other to drive neoplastic processes, a number of in vitro and ex vivo mechanisms support the role of platelets in mediating tumor survival, proliferation, and chemoresistance; these likely underlie the epidemiologic associations with patient survival. In vivo mouse models have validated these results, demonstrating reductions in tumor growth with anti-platelet therapy, and prospective and retrospective clinical data evaluating direct or indirect anti-platelet agents suggest that anti-platelet therapy may provide a novel therapeutic strategy in ovarian cancer. Clinical trials are underway to evaluate therapies with anti-platelet activity, such as aspirin and metformin, and these will provide further insight into potential therapeutic targets [40,106,107]. Available evidence strongly suggests that pretreatment thrombocytosis is an independent prognostic factor for ovarian cancer and that monitoring platelet counts regularly during and after therapy may be a possible avenue to assess response to therapy, disease progression, or recurrence. Future studies should seek to augment our understanding of associations of thrombocytosis with ovarian cancer prognosis to identify potential novel treatment strategies.

## Figures and Tables

**Table 1:** Characteristics of Studies on Thrombocytosis and Ovarian Cancer Outcomes.

Author	Year	Platelet Measurement Timeframe(s)			Thrombocytosis Threshold(s)	Study Population			Statistical Analysis		Prognostic Association(s) *	
		Description	Pretreatment	Additional		Patients	Description	Source (Country)	Outcome	Approach	PFS/DFS	OS/DSS
Allensworth	2013	Preoperative (NOS)	Yes		450	578	epithelial ovarian cancer	United States	DFS, OS	KM, PHreg	Worse (unadj, adj)	Worse * (unadj)
Andersen	2014	Up to 3-years prior to diagnosis	Yes		400 (mild), 550 (severe)	224	ovarian cancer (NOS)	Denmark	All-Cause and Cancer-Specific Mortality	KM, PHreg	Worse (unadj, adj)	
Barber	2015	Preoperative (NOS)	Yes		450	1072	ovarian cancer (NOS)	ACS NSQIP (US)	30-Day Outcomes	Logistic Regression	Worse for major complications (unadj, adj)	
Bottsford-Miller	2015	Pretreatment (at diagnosis) and at disease recurrence	Yes	Yes	450	341	recurrent epithelial ovarian cancer	United States	PFS, OS	KM	NS (P = 0.05)	Worse
Bozkurt	2004	Second-look laparotomy after chemotherapy (up to 5 days prior)		Yes	380 (ROC), continuous	37	advanced stage (III/IV) epithelial ovarian cancer	Turkey	Presence of Disease	Wilcoxon Signed Rank Test	Worse (P = 0.025)	
Canzler	2020	Pretreatment for recurrent disease		Yes	400	300	recurrent epithelial ovarian cancer	Germany	PFS, OS	KM, PHreg	NS (unadj)	Worse * (unadj)
Chen, JP	2019	Pretreatment (NOS)	Yes		400	108	advanced stage (IV) epithelial ovarian cancer	China	PFS, OS	KM, PHreg	NS (KM), Worse in combination with CA-125 (unadj, adj)	Worse (KM), Worse in combination with CA-125 (unadj, adj)
Chen, Y	2015	Pretreatment (NOS)	Yes		400	816	epithelial ovarian cancer	China	PFS, OS	KM, PHreg	Worse (unadj, adj)	Worse (unadj, adj)
Cohen	2014	Cytoreductive surgery for recurrent disease		Yes	350	107	recurrent epithelial ovarian cancer	United States	OS	KM, PHreg		Worse (unadj, adj)
Cozzi	2016	Date of diagnosis and up to 1, 2, 4, and 8 weeks prior	Yes		350, 400, 450	304	epithelial ovarian cancer	United States	OS	KM, PHreg		Worse (unadj, adj)
Digklia	2014	Pretreatment (at diagnosis)	Yes		350	91	stage III/IV serous ovarian cancer	Switzerland	PFS, OS	KM, PHreg	Worse (unadj, adj)	Worse (unadj, adj)
Eggemann	2015	At diagnosis, after surgery, before and after chemotherapy, and disease recurrence	Yes	Yes	350	132	ovarian cancer (NOS)	Germany	PFS, OS	KM, PHreg	Worse for <25% reduction (unadj, adj)	Worse for <25% reduction (unadj, adj)
Feng	2016	Preoperative (NOS)	Yes		450	874	high-grade serous ovarian cancer	China	PFS, OS	KM	NS	NS
Gerestein	2009	Preoperative (within 1 week of surgery)	Yes		continuous	118	advanced stage (IIB-IV) epithelial ovarian cancer	The Netherlands	PFS, OS	KM, PHreg	Worse (unadj, adj)	Worse (unadj, adj)
Gungor	2009	Preoperative (within 14 days of surgery)	Yes		400	292	epithelial ovarian cancer	Turkey	OS	KM, PHreg		Worse (unadj, adj)
Hefler-Frischmuth	2018	Preoperative (24–72 h prior to initial surgery)	Yes		450, continuous	498	epithelial ovarian cancer	Austria	OS	KM, PHreg		Worse * (unadj)
Hu	2020	Pretreatment, 14 days after chemotherapy, and disease recurrence	Yes	Yes	300	104	recurrent epithelial ovarian cancer	China	PFS, OS	KM, PHreg	Worse (unadj, adj)	Worse (unadj, adj)
Komura	2019	Lowest measure between diagnosis and treatment	Yes		427 (ROC)	308	epithelial ovarian cancer	Japan	DSS	KM, PHreg		Worse (unadj, adj)
Lee	2011	Preoperative (within 7 days prior to surgery) and after adjuvant chemotherapy	Yes	Yes	400	179	advanced stage (III/IV) epithelial ovarian cancer	Korea	PFS, OS	KM, PHreg	NS (unadj)	Worse (unadj, adj)
Li	2004	Preoperative (within 14 days of surgery)	Yes		400	144	advanced stage (III/IV) epithelial ovarian cancer	United States	DFS, OS	KM, PHreg	Worse (unadj, adj)	Worse (unadj, adj)
Ma	2013	Preoperative (within 7 days prior to surgery)	Yes		400	182	epithelial ovarian cancer	China	PFS, OS	KM, PHreg	Worse in combination with MAR (unadj, adj)	Worse in combination with MAR (unadj, adj)
Man	2015	Pretreatment (up to 7 days prior)	Yes		300	190	epithelial ovarian cancer	China	PFS, OS (3-year)	KM, PHreg	Worse (unadj, adj)	Worse (unadj, adj)
Matsuo	2015	At diagnosis and at disease progression or recurrence	Yes	Yes	400	1308	clear cell and serous ovarian cancer	10 academic institutions (US, Japan, England)	PFS, OS	KM, PHreg	Worse (unadj, adj)	Worse (unadj, adj)
Menczer	1998	Preoperative (NOS)	Yes		400	70	epithelial ovarian cancer	Israel	OS	KM		Worse
Nakao	2020	Pretreatment (mean of initial and pre-treatment evaluations)	Yes		400	280	epithelial ovarian cancer	Japan	PFS, OS	KM, PHreg	Worse (unadj, adj)	Worse (unadj, adj)
Okunade	2020	Pretreatment (at diagnosis)	Yes		450	72	epithelial ovarian cancer	Nigeria	PFS, OS (3-year)	KM, PHreg	Worse (unadj, adj)	Worse (unadj, adj)
Qiu	2012	Preoperative (2–4 days prior)	Yes		400	136	epithelial ovarian cancer	China	PFS, OS	KM, PHreg	Worse * (unadj)	Worse * (unadj)
Słabuszewska-Jóźwiak	2015	Preoperative (1 day before surgery)	Yes		350	97	ovarian cancer (not all epithelial)	Poland	DFS, OS	Mann-Whitney U Test	NS	Worse
Soonthornthum	2007	Preoperative (within 14 days of surgery)	Yes		305 (ROC), 400	74	epithelial ovarian cancer	Thailand	OS	KM		Worse
Stone	2012	Preoperative (NOS)	Yes		450	619	epithelial ovarian cancer	United States	PFS, OS	KM, PHreg	Worse (unadj)	Worse (unadj, adj)
Tang	2017	Preoperative (NOS)	Yes		300, 327 (ROC), 350, 400	171	epithelial ovarian cancer	China	OS	KM		Worse
Zeimet	1994	Preoperative (NOS)	Yes		400	130	epithelial ovarian cancer	Austria	OS (4-year)	KM		NS

Abbreviations: adj—adjusted; DFS—disease-free survival; DSS—disease-specific survival; KM—Kaplan–Meier analysis; MAR—Mean Aggregation Rate; NOS—not otherwise specified; NS—not significant; OS—overall survival; P—*p*-value; PFS—progression-free survival; PHreg—proportional hazards regression; ROC—receiver operating curve; unadj—unadjusted; US—United States. * Denotes that adjusted proportional hazards regression model was not significant.
